# Spatial distribution of urogenital schistosomiasis in school-aged children in Togo: an oversampling survey in three districts in 2022

**DOI:** 10.1186/s13071-026-07357-6

**Published:** 2026-03-18

**Authors:** Essoham Ataba, Fiali Ayawa Lack, Hélène Eya Kamassa, Smaila Alidou, Kossi Yakpa, Fiona M. Fleming, Efoe Sossou, Manani Hemou, Mawèké Tchalim, Gbati Datagni, Anders Seim, Piham Gnossike, Penelope Vounatsou, Rachel Pullan, Katherine Gass, Ameyo M. Dorkenoo

**Affiliations:** 1 National Malaria Control Program, Ministry of Health and Public Hygiene, Lomé, Togo; 2 Centre Hospitalier Universitaire Sylvanus Olympio, Ministry of Health and Public Hygiene, Lomé, Togo; 3 Laboratoire de Microbiologie et de Contrôle de Qualité des Denrées Alimentaires, Unité de Recherche en Immunologie et Immunomodulation (UR2IM), Ecole Supérieure des Techniques Biologiques et Alimentaires (ESTBA), Université de Lomé, Lomé, Togo; 4 West African Centre for Cell Biology of Infectious Pathogens (WACCBIP), University of Ghana, Accra, Ghana; 5 Department of Biochemistry, Cell and Molecular Biology, College of Basic and Applied Sciences, University of Ghana, Accra, Ghana; 6 Département de Santé Publique, Université, Unité de Formation et de Recherche en Sciences de la Santé, Joseph KI-ZERBO, Ouagadougou, Burkina Faso; 7 Department of Social and Preventive Medicine, Université de Montréal, Montreal, Canada; 8 Unlimit Health, London, UK; 9 Ministry of Health and Public Hygiene, Lomé, Togo; 10 Health and Development International, Représentation, Lomé, Togo; 11 Health and Development International, Fjellstrand, Norway; 12 Swiss Tropical and Public Health Institute, Basel, Switzerland; 13 London School of Tropical Medicine and Hygiene, London, UK; 14 Task Force for Global Health, Decatur, GA USA; 15 Department of Biological and Basic Sciences, Faculty of Health Sciences, University of Lomé, Lomé, Togo

**Keywords:** Oversampling, Urogenital schistosomiasis, Spatial distribution, 2022, Togo

## Abstract

**Background:**

Despite concerted global efforts, urogenital schistosomiasis remains an important public health challenge in sub-Saharan Africa. Understanding the local dynamics of this disease is crucial for effectively planning interventions to eliminate urogenital schistosomiasis as a public health concern. This study, conducted in southern Togo, aimed to comprehensively describe the epidemiology of *Schistosoma haematobium* infection among school-aged children after more than a decade of control using preventive chemotherapy.

**Methods:**

A cross-sectional study was conducted from July to August 2022 among children aged 5 to 14 years in selected villages across three districts of the Plateaux region of Togo. Coordinates of each surveyed household were recorded, and sociodemographic data were gathered using a pre-tested questionnaire. Urine samples were visually examined for macrohematuria and tested for microhematuria. Urine filtration was performed to quantify *S. haematobium* eggs.

**Results:**

In total, 3146 households were enrolled, and 6400 school-aged children provided urine samples. The overall prevalence of macrohematuria was 4.48% (95% CI [4.21–4.75]) and of microhematuria was 16.11% (95% CI [15.65–16.57]), while 15.0% (95% CI [14.55–15.45]), of children were *S. haematobium* egg-positive. Prevalence varied across villages and districts, with Ogou district exhibiting the highest prevalence by all three indicators. The prevalence of heavy-intensity infection ranged from 0.92% (95% CI [0.69–1.15]) in the Est-Mono district to 12.91% (95% CI [12.3–13.52]) in the Ogou district, averaging 3.40% (95% CI [2.95–3.85]) in the Anié district.

**Conclusions:**

This study highlights geographic variability in the distribution of *S. haematobium*, even after more than a decade of PC. The detailed data provided can support the development of more targeted treatment strategies, focusing on areas where *S. haematobium* remains prevalent. This approach may accelerate progress toward achieving the WHO goal of eliminating schistosomiasis as a public health concern.

**Graphical Abstract:**

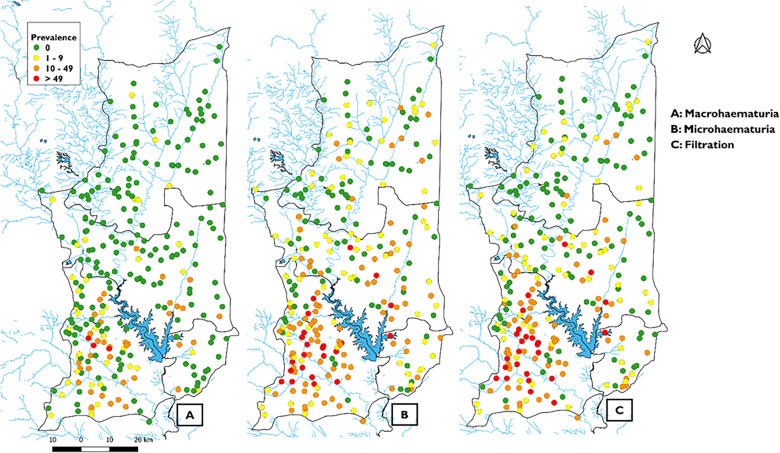

## Background

Schistosomiasis (SCH) is a parasitic disease in humans caused by trematode worms of the genus *Schistosoma* [1]. Among the six species known to infect humans, two are particularly prevalent in sub-Saharan Africa: *Schistosoma haematobium*, which causes urogenital schistosomiasis, and *S. mansoni*, responsible for intestinal schistosomiasis. These infections pose significant public health challenges in the region, leading to chronic illness and impairing the quality of life of millions of individuals. Schistosomiasis is classified as one of the neglected tropical diseases (NTDs) because of its widespread impact and the relative lack of attention and resources devoted to its control and treatment [2–4]. Preventive chemotherapy (PC) continues to be the cornerstone of the World Health Organization (WHO)-proposed approach to reduce the burden of this disease through the mass distribution of praziquantel to populations at risk. Over the past 2 decades, most endemic countries have successfully scaled up PC using school or community-based distribution platforms in districts with moderate to high schistosomiasis prevalence [5, 6]. In Togo, the schistosomiasis control program was initiated in 2010 following baseline mapping to identify regions requiring PC. This PC strategy was implemented through community-based, door-to-door mass drug distribution [7]. In line with WHO recommendations, several surveys have been carried out, including the baseline mapping in 2009, and the impact assessments in 2015 and 2021, following the annual praziquantel distribution cycles [8]. The 2009 and 2015 surveys were carried out at a fine spatial resolution covering the whole country and sampled two villages per Peripheral Health Unit (PHU), the administrative level below the district used to implement praziquantel distribution. This meticulous approach allowed for precise monitoring and evaluation of the program's effectiveness in reducing schistosomiasis prevalence and intensity. This strategy enabled the Togo Ministry of Health to understand the spatial distribution and infection levels within endemic areas, which was instrumental in determining the optimal frequency of drug distribution, but also for identifying the specific groups that should be targeted.

WHO has shifted its focus from morbidity control to the elimination of schistosomiasis as a public health concern by 2030 [7, 8]. As such, there is a growing need to develop standardized approaches to measure the impact of PC on schistosomiasis prevalence and reevaluate the appropriate geographic scale at which to target PC. To support this transition, a large, multi-country study, for which Togo was one of four country sites, was launched to gather high-quality, high-spatial-resolution data on the distribution of schistosomiasis after several years of control efforts [9]. The purpose of this study (referred to as the “Schistosomiasis Oversampling Study”) was to collect fine-scale data on the spatial distribution from diverse epidemiological settings, which could be used to generate ‘truth surfaces’ upon which simulations could be run to identify the optimal sampling strategy for impact assessments. This study provides comprehensive epidemiological data on the current prevalence and intensity of schistosomiasis in the Plateaux region, a major endemic hotspot for *S. haematobium* in Togo.

## Methods

### Study setting

**Fig. 1:**
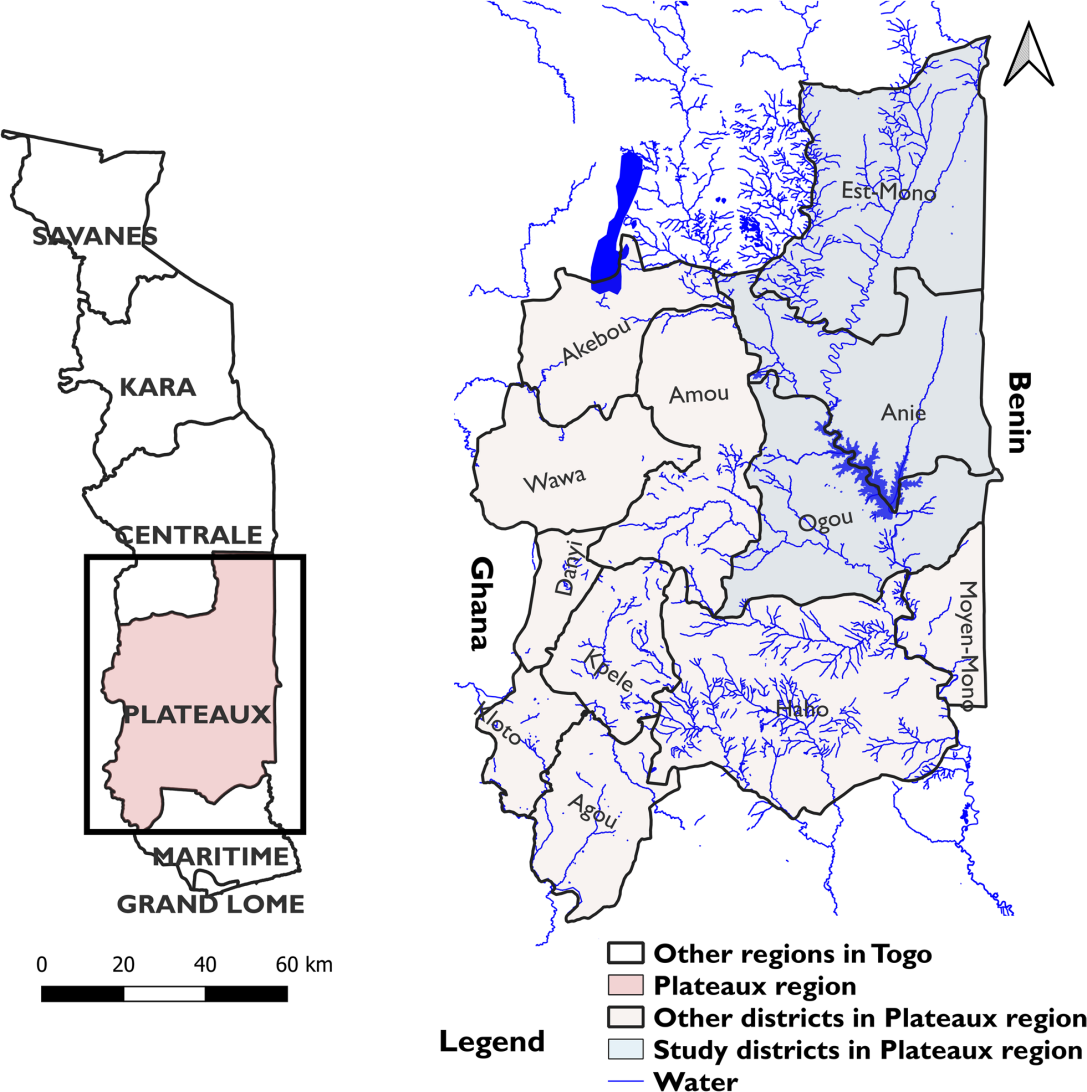
Study area map. Location of the three selected districts (Ogou, Anié, Est-Mono) in the Plateaux region, Togo. Shapefiles obtained from Humanitarian Data Exchange (data.humdata.org); map created with QGIS 2.18 "Las Palmas"

This study employed a cross-sectional approach to survey three districts within the Plateaux health region of Togo from July 11 to August 11, 2022. Togo was selected for this multi-country study to represent the archetypes of small perennial bodies of water and artificial lakes, specifically the districts of Est-Mono, Anié, and Ogou in the Plateaux region (Fig. 1).

These districts feature diverse environmental conditions, thereby enhancing the study's ability to capture the full spectrum of schistosomiasis transmission dynamics and inform tailored intervention strategies. Additional criteria for site selection included a *S. haematobium* infection prevalence > 5% in the previous assessment. Collectively, these three districts cover an area of 7701 km². As of 2022, they have an estimated population of 571,073. The districts of Est-Mono, Anié, and Ogou are recognized as priority areas in Togo due to the high prevalence of schistosomiasis identified through baseline studies conducted in 1999 [12]. To this end, the first mass treatment campaigns (with praziquantel) targeting school-aged children were launched in 2000 with limited geographic coverage. In 2007, a pilot integrated mapping for schistosomiasis, soil-transmitted helminthiasis (STH), and trachoma was carried out in the Binah district, followed by a nationwide integrated mapping in 2009 [13]. Given the prevalence of these three diseases, Togo initiated its integrated preventive chemotherapy program to combat NTDs in 2010, using community mass drug administration (MDA). Between 2010 and 2020, these PC interventions were intensified with annual or biannual treatment campaigns. Ivermectin administration for onchocerciasis control, which began in 1988 following baseline assessment, has been integrated with praziquantel and albendazole distribution since 2010 to target schistosomiasis, STH, and onchocerciasis, respectively, as part of a coordinated PC-NTD control strategy. Drug distribution was conducted door-to-door at the household level, and the number of annual treatment rounds for schistosomiasis varies by primary health unit (PHU), based on local prevalence levels [8, 14, 15].

### Study design and sample size

Eligible participants were children between the ages of 5 to 14 years, living in a selected household, who had written parental or guardian consent, provided assent to participate, and were able to provide a urine sample.

The primary sampling unit (PSU) was defined as a village. Local authorities provided a complete list of villages in the three selected districts, along with their GPS coordinates and 2021 population estimate. Of these PSUs, 40% were randomly selected for inclusion in the study, corresponding to 273 PSUs. Approximately 36 school-aged children (SAC) were enrolled per selected PSU. This sample size, accounting for an anticipated non-response rate of 20%, was deemed sufficient to estimate the prevalence of *Schistosoma* infection in the area.

### Sampling design

#### PSU and segment selection

Enrollment of individuals was performed using modified compact segment sampling [10], with each selected PSU divided into segments of approximately 50 households. The number of segments per village was predetermined before the visit, based on the village population and average household size in Togo (5.1 individuals). Upon arrival in a selected village, the survey team subdivided the village into the predetermined number of segments with the support of village chiefs and community health workers (CHWs), using natural boundaries, such as roads, paths, streams, or other identifiable landmarks. Each segment was assigned a number, and one segment was randomly selected. In villages with < 30 households, the entire village was considered a single segment and subsequently enrolled in the study.

#### Households and SAC selection

For the household selection, after all households in had been numbered, a sampling list was used to select the households within the segment. A predefined sampling list was used to determine which households within the segment would be selected. To reduce the risks of surveyor bias and to better meet sample size targets, two household sampling lists (“List A” and “List B”) were created for each district. These within-segment sampling lists were generated separately for each district, based on the average household size and anticipated non-response rate, to reach the target sample size per PHU. In selected segments with ≤ 30 households, all school-aged children (SAC) residing in selected households that met the inclusion criteria were invited to participate. To gather a high rate of household adherence, the survey teams, with the support of PHU managers and CHWs, informed village leaders several days before their arrival. The day before the survey, the information was disseminated throughout the community by the town crier.

#### Data collection

The sociodemographic data and biological results were reported via an electronic questionnaire on the Secure Data Kit (SDK) platform managed by Standard Data. The GPS coordinates of each surveyed household and village were collected. Information regarding drinking water, hygiene, and sanitation (WASH), the availability of schooling for children, and the economic level of household heads was also collected. Here, we focus only on the spatial dynamics of the prevalence among these three districts. The data were exported to the Standard Data online platform for monitoring; hardcopy backup forms were also collected to ensure accuracy of the data provided to the SDK tool.

#### Urine sample collection

The urine sample was provided by each enrolled SAC in an empty, clean, labeled container after he or she had been instructed about proper hygienic practices for sample collection.

#### Biological testing

Visual examination of urine samples was performed to detect macrohematuria. Dipsticks (Haemastix^©^) (Siemens Healthcare Diagnostics GmbH; Eschborn, Germany) were used to detect microhematuria as a proxy of the presence of *S. haematobium* in the urine sample. This result was recorded and graded within 60 s according to the manufacturer’s guidelines. The various observable results were as follows: negative (no reaction), trace (non-hemolyzed blood), trace (hemolyzed blood), + (approximately 25 erythrocytes/µl), + + (approximately 80 erythrocytes/µl), + + + (approximately 200 erythrocytes/µl). The last four results categories were considered positive [11].

Urine filtration was performed for egg examination and quantification. Urogenital schistosomiasis infection was defined by the presence of hematuria and/or *S. haematobium/S. mansoni* eggs in the urine [8]. Infection was categorized according to the WHO guidelines, where heavy-intensity infections were defined as ≥ 50 eggs per 10 ml urine [12].

### Statistical analysis

Data were processed and analyzed using Stata 16 (StataCorp, College Station, TX, USA); maps were generated using QGIS 3.01 software (Girona). Households without a child who provided a urine sample were excluded from the final dataset. Categorical variables were expressed as proportions, and quantitative variables were expressed as maximum and minimum or as means with standard deviation (SD). Pearson correlation was used to assess the correlation between the prevalence and the intensity of *S. haematobium* infection.

## Results

### General characteristics of the study population

**Fig. 2:**
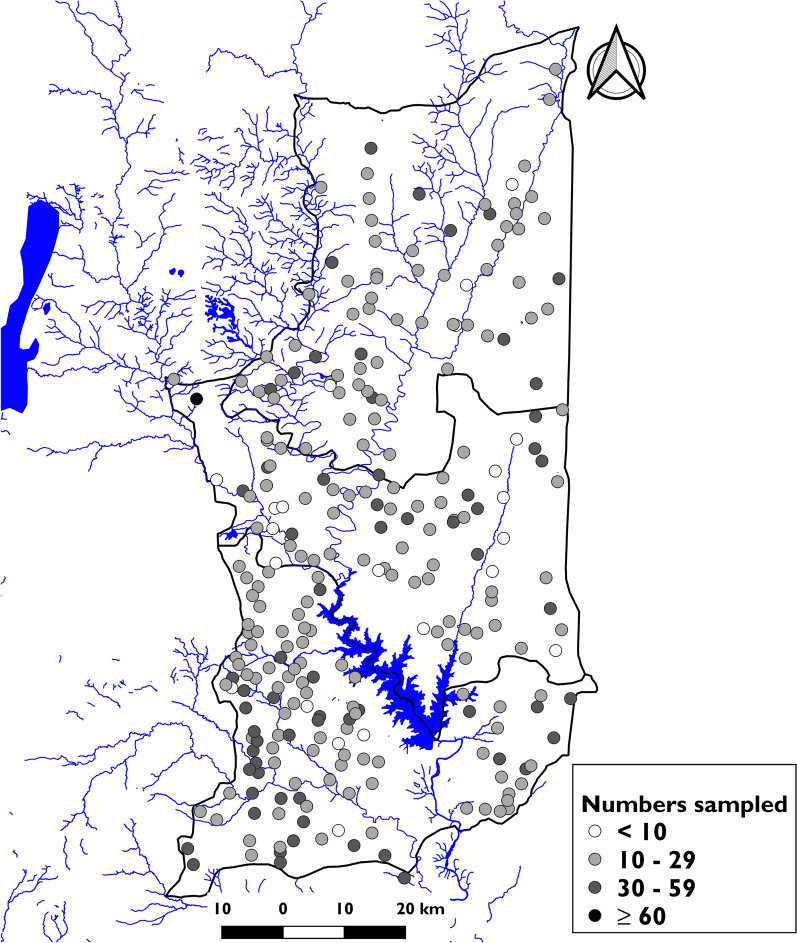
Numbers of SAC sampled by village. Shapefiles obtained from Humanitarian Data Exchange (data.humdata.org); map created with QGIS 2.18 "Las Palmas"

A total of 5908 households, including 1686 in Anié, 1677 in Est-Mono, and 2545 in Ogou, were surveyed in 270 villages across 46 PHUs in the three selected districts. Approximately 73% (4302/5908) of these households were interviewed, and 3146 (73.1%) included members of the target population (SAC), of whom 97.3% were successfully enrolled. Six thousand four hundred (6400) (94.5%) of these SAC provided urine samples (Table 1). The flow diagram was previously described [9], and the median number of SAC included per village was 23 (16–30). Furthermore, the distribution of SAC by village varied, with some PSUs enrolling < 10 children (Fig. 2).

**Table 1:** Characteristics of surveyed households by district in the Plateaux region, Togo

Health district	Anié	Est-Mono	Ogou	Total
Total number of PHUs/district n	14	19	14	47
Number of PHUs surveyed (%)	13 (92.2)	19 (100.0)	14 (100)	46 (97.8)
Number of villages surveyed	78	74	118	270
Number of households visited	1686	1677	2545	5908
Number of households surveyed (%)	1132 (67.1)	1212 (72.3)	1958 (76.9)	4302 (72.8)
Number of households surveyed with SAC present (%)	810 (71.6)	891 (73.5)	1445 (73.8)	3146 (73.1)
Number of consenting households (%)	800 (98.8)	883 (99.1)	1427 (98.8)	3110 (98.9)
SAC present	1798	1883	3284	6965
SAC with assent (%)	1739 (97.7)	1801 (95.6)	3234 (98.4)	6774 (97.3)
Assenting SAC who provided samples (%)	1647 (94.7)	1732 (96.2)	3021 (93.4)	6400 (94.5)

### Prevalence of urogenital schistosomiasis

The prevalence of urogenital schistosomiasis varied by diagnostic methodology, surveyed village, and district. The mean prevalence of macrohematuria was 4.48% (95% CI [4.21–4.57]), of microhematuria 16.11% (95% CI [15.65–16.57]), and of filtration 15.0% (95% CI [14.55–15.45]). At the PHU level, prevalence ranged from 0% to 44.14% (Table 2).

**Table 2:** Prevalence of *Schistosoma haematobium* by diagnostic method and district in the Plateaux region, Togo, 2022

	Anié	Est-Mono	Ogou	Total
*Schistosoma haematobium* prevalence				
District-level mean (SE)	7.53 (0.65)	2.08 (0.34)	26.36 (0.80)	15.0 (0.45)
Median and range by PHU	5.43 (2.04–15.82)	1.29 (0–7.45)	21.67 (0.70–44.14)	3.88 (0–44.14)
Median and range by village	2.91 (0–69.70)	0 (0–42.86)	15.96 (0–100)	4.35 (0–100)
Prevalence of heavy-intensity infection				
District-level mean (SE)	3.40 (0.45)	0.92 (0.23)	12.91 (0.61)	7.22 (0.32)
Median and range by PHU	0.28 (0–12.03)	0.05 (0–4.35)	0.88 (0.70—26.17)	0.16 (0–26.17)
Median and range by village	0.04 (0–51.51)	0.01 (0–25.00)	0.11 (0–81.81)	0.03 (0.0–81.82)
Prevalence of macrohematuria				
District-level mean (SE)	2.37 (0.37)	0.29 (0.13)	8.81 (0.51)	4.48 (0.27)
Median and range by PHU	0.19 (0–12.41)	0.02 (0–1.24)	0.63 (0–26.64)	0.11 (0.0–26.64)
Median and range by village	0.03 (0–33.33)	0.00 (0–4.5)	0.07 (0–90.91)	0.02 (0.00–90.91)
Prevalence of microhematuria infection				
District-level mean (SE)	10.81 (0.77)	3.70 (0.45)	26.12 (0.80)	16.11 (0.46)
Median and range by PHU	0.90 (2.04–22.76)	0.21 (0–12.36)	1.87 (4.17–40.74)	0.37 (0.0–40.74)
Median and range by village	0.14 (0–69.69)	0.05 (0–42.95)	0.22 (0–96.66)	0.06 (0.0–96.67)

**Fig. 3:**
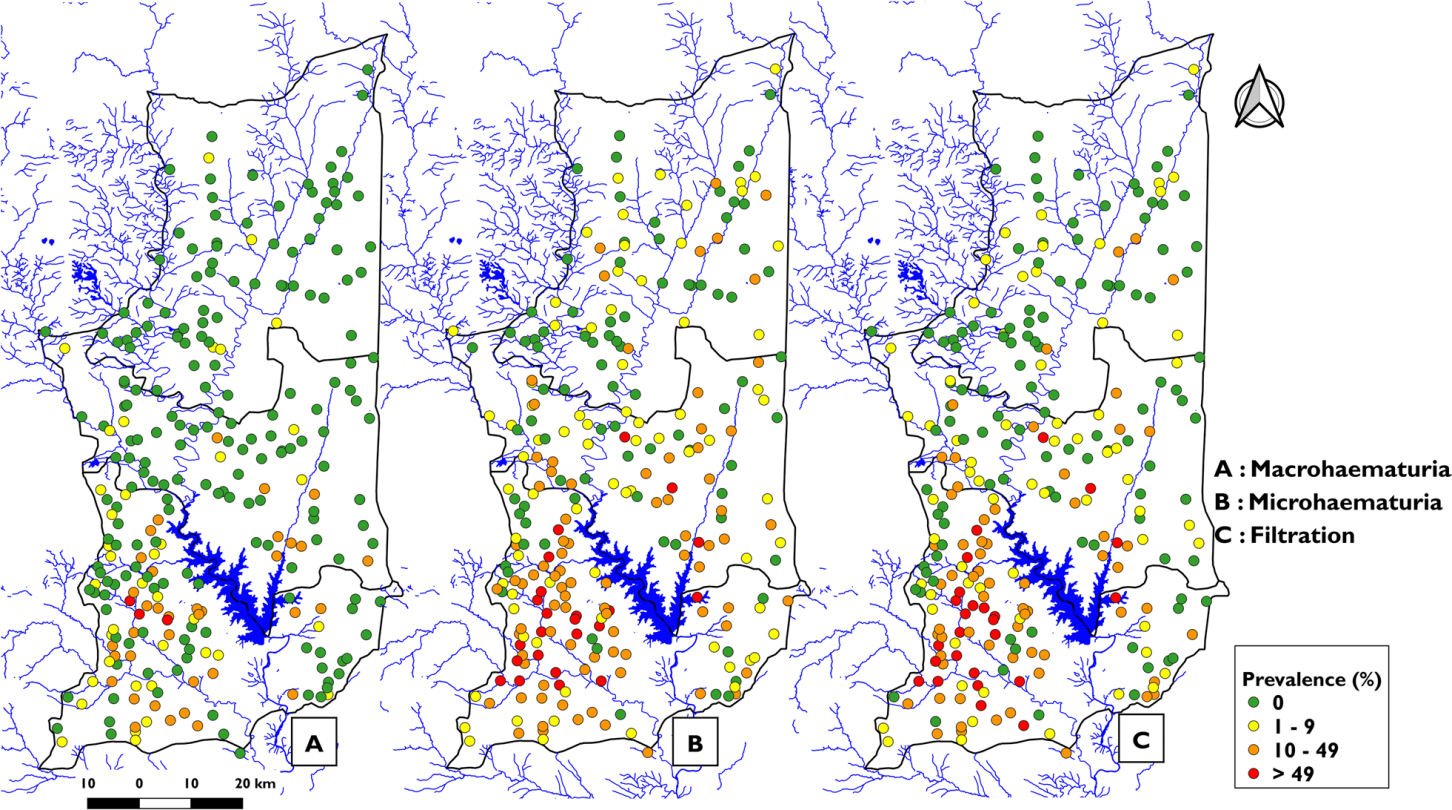
Prevalence of urogenital schistosomiasis by village, in the Plateaux region, Togo, 2022. **A** Distribution by macrohematuria. **B** Distribution by dipstick (microhematuria). **C** Distribution by urine filtration. Shapefiles obtained from Humanitarian Data Exchange (data.humdata.org); map created with QGIS 2.18 "Las Palmas"

The distribution of prevalence per village showed a higher prevalence in Ogou district for all three methods. In this district, the prevalence observed in villages varied from 0 to 100%, with a median prevalence of 16%. Approximately 41.68% of villages had a prevalence between 0 and 10%. Moving from the Ogou district to Est-Mono, the village-level prevalences decreased, with many villages showing low (< 10%) or zero prevalence, regardless of the diagnostic approach used (Fig. 3).

### Intensity and distribution of urogenital schistosomiasis

**Fig. 4:**
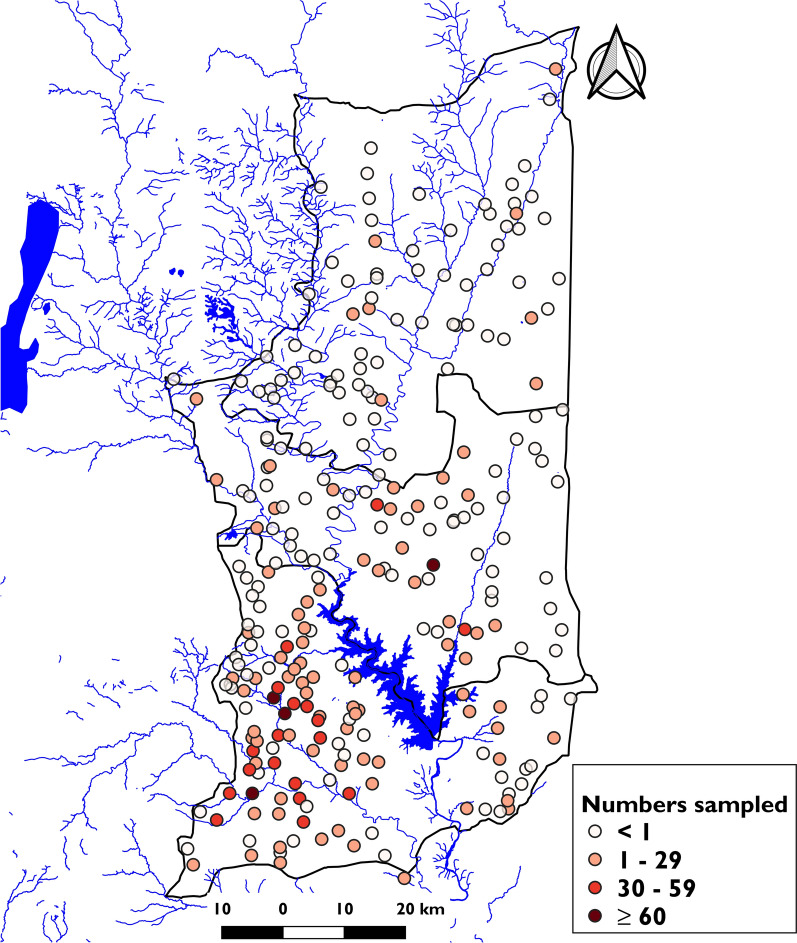
Distribution of the prevalence of heavy-intensity urogenital schistosomiasis infections by surveyed village in the Plateaux region, Togo, 2022. Shapefiles obtained from Humanitarian Data Exchange (data.humdata.org); map created with QGIS 2.18 "Las Palmas"

**Fig. 5:**
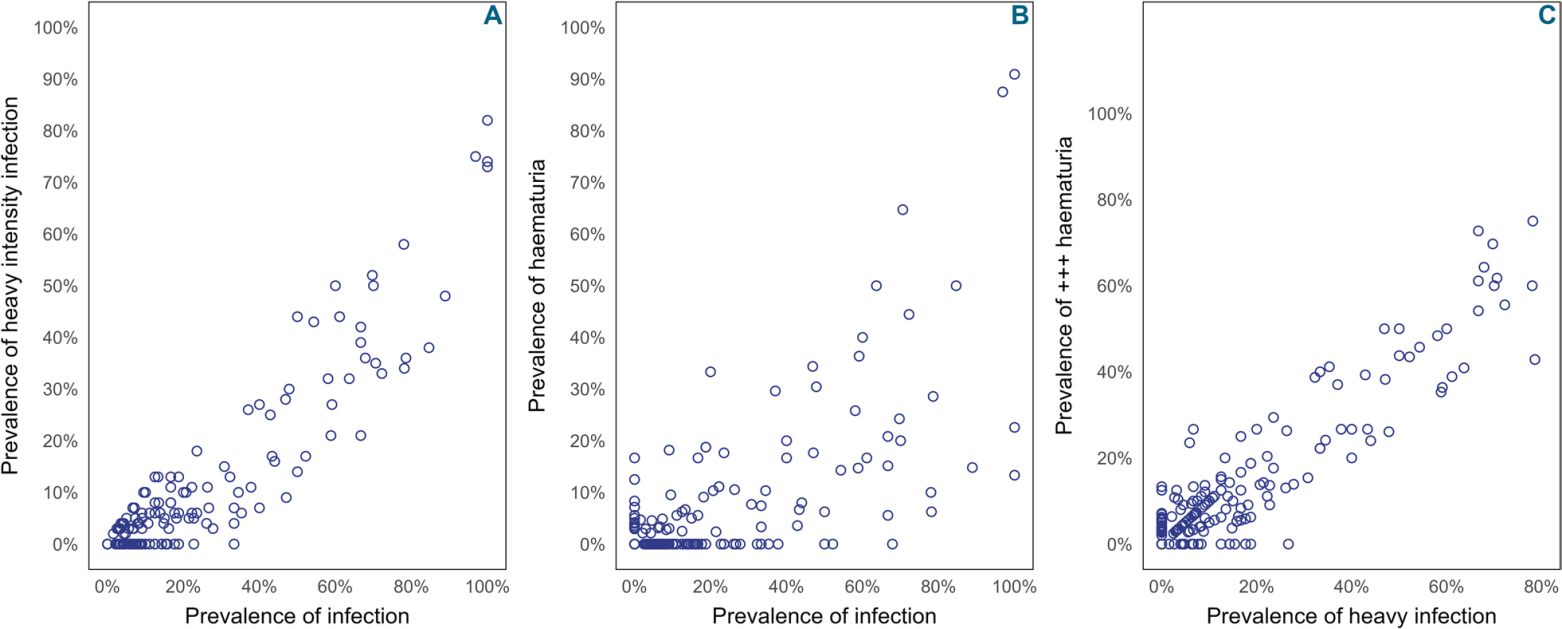
Relationship between infection prevalence and morbidity indicators at the village level. Prevalence of **A** heavy-intensity *Schistosoma haematobium* infection vs any infection [pairwise correlation, *r* = 0.92 (*p* < 0.001)]; **B** microhematuria (trace with hemolyzed blood and above) vs any infection [pairwise correlation, *r* = 0.95 (*p* < 0.001)]; **C** microhematuria (+++) vs heavy-intensity *S. haematobium* infection [pairwise correlation, *r* = 0.95 (*p* < 0.001)]

The mean prevalence of heavy-intensity schistosomiasis infection was 0.92% (95% CI [0.69–1.15]) in Est-Mono district, 3.40% (95% CI [2.95–3.85]) in Anié district, and 12.91% (95% CI [12.3–13.52]) in Ogou district (Table 2). The geospatial distribution of heavy-intensity infection prevalence is illustrated in Fig. 4, where it is apparent that the heaviest intensity is in Ogou district, followed by decreasing intensity in the Anié and Est Mono districts. The correlation between the prevalence of infection (by urine filtration) and the prevalence of heavy-intensity infection at the village level was substantial (*r* = 0.92, *p* < 0.001) (Fig. 5 A). The prevalence of any level of microhematuria was strongly correlated with the prevalence of infection by urine filtration (*r* = 0.95, *p* < 0.001) (Fig. 5 B), while the prevalence of microhematuria (+ + +) was only strongly correlated with the proportion of heavy-intensity infection (*r* = 0.95, *p* < 0.001) (Fig. 5 C).

The median prevalence of heavy-intensity infection by PHU ranged from 0.05% [0% to 4.35%] in the Est-Mono district to 8.08% in the Ogou district with a range of [0.70–26.17] (Table 2). However, the distribution of the prevalence of heavy-intensity infections by site shows that most sites in the Est-Mono and Anié districts have a < 1% prevalence of heavy-intensity infection, while Ogou district has many PSUs with > 10% heavy-intensity infections (Fig. 4).

## Discussion

This study, conducted in three districts (Est-Mono, Anié, and Ogou) of the Plateaux region in Togo, reports the prevalence and spatial distribution of *S. haematobium* infection in school-aged children from a population that has received 12 years of community-based PC with praziquantel. As part of a multi-country study to generate fine-scale epidemiological surfaces of schistosomiasis prevalence, this study is one of the first conducted in Togo to have such granular data, allowing for a more reliable epidemiological understanding of this disease.

This study revealed marked geographical heterogeneity in the distribution of urogenital schistosomiasis across the three districts in Togo. Est-Mono reported the lowest mean prevalence (2.08%), followed by Anié (7.53%) and Ogou, which recorded the highest (26.36%). However, this variation in average prevalence at the district level conceals substantial heterogeneity between villages. For example, in the Ogou district, village-level prevalence ranges from 0 to 100%, with a median of 16%. Approximately 41.68% of villages had a prevalence of 0–10%.

This suggests that a treatment decision at the district level should consider village-level prevalence to avoid unnecessary drug distribution. However, the decision taken by Togo to carry out MDA based on the prevalence of PSU instead of districts as recommended by the WHO was salutary, but it must be reinforced, if possible, to go to the village level and adapt this treatment to the epidemiological situation of each village; however this risks increasing the cost of the intervention in countries that often do not have the domestic resources to implement these control actions. The maintenance of the transmission cycle could be partly favored by several determinants, particularly in the Ogou district [9]. Indeed, the large Mono water dam in this district brings together several water-related commercial activities [13, 14]. In this area, the quality of water distribution could also be a factor [15, 16]. Relevant socioeconomic factors include limited access to safe water and sanitation facilities, reliance on surface water for domestic and occupational activities (such as farming, fishing, and laundry), low household income, low educational attainment, and inadequate health-seeking behaviors, all of which may increase exposure to *S. haematobium*-infested water [9]. Overall, the distribution of *S. haematobium* is often linked to the ecological behaviors and preferences of the snails, *Bulinus* spp.[17–19], whose ability to hibernate during dry seasons allows them to colonize seasonal water bodies [17, 19, 20]. Further studies will enable us to assess the reasons for the persistence of the high urinary schistosomiasis prevalence in this area.

The overall prevalence of 16.11% microhematuria in the present study, conducted in 2022, is higher than expected, based on findings from the 2015 impact assessment survey [21, 22], which reported a reduction in microhematuria prevalence in the Plateaux region from 23.9% in 2009 [21] to 6.2% in 2015 [22]. This increase in prevalence in 2022 could be because the number of villages surveyed is higher and more representative, especially since this survey was community-based, unlike the 2015 survey, where the SAC were enrolled in schools.

It is also encouraging to note the strong correlation between the prevalence of infection, as measured by urine filtration, and microhematuria. The equally strong correlation between heavy-intensity infections and the high positivity (+++) of microhematuria suggests that there may be situations where the results of a dipstick examination can replace urine filtration, given the relative ease of the dipstick tool compared to filtration [23–25], especially in the current context of scarce financial resources to support impact assessment activities.

After 12 years of several rounds of community-based PC, we would have expected to see further reductions in the prevalence of schistosomiasis by 2022, but this study has revealed hidden pockets of infection that were not identified in previous assessments. Additionally, the use of community sampling, carried out at the household level and mobilizing a larger number of participants, includes children who are absent or out of the school system, which greatly reduces the coverage bias present in school surveys; this makes it possible to obtain data that are more representative of the reality of endemicity in the three districts [20]. Indeed, in previous evaluations in Togo, children were enrolled in school [21, 22, 26], and the number of children surveyed per PHU was relatively lower. The prevalence obtained in this current study is thus more representative of the current situation in the survey area, given the high proportion of villages surveyed per district (around 40% of villages) compared to the two previous surveys.

The oversampling survey enabled us to go beyond the usual approach using urine strips. Systematic filtration of urine samples enabled us to document the parasite load more precisely, and the results confirmed a high degree of concordance between overall prevalence and that of heavy-intensity infections. Indeed, the intensity of *S. haematobium* infection follows the same trend seen in the prevalence data, with Ogou district recording the highest prevalence of heavy-intensity infections, varying from 0 to 81.81% depending on the villages surveyed. Similarly, the median prevalence of schistosomiasis per health district followed the same trend as the intensity of urogenital schistosomiasis per district, with Ogou district having the highest median prevalence at both the village and health unit levels.

The oversampling approach employed in this study revealed both heterogeneity and homogeneity in the distribution of urogenital schistosomiasis within and between districts. These findings directly align with the WHO 2030 NTD Roadmap, which emphasizes the importance of granular epidemiological data to guide the transition from uniform to more targeted PC strategies in endemic settings [27]. By demonstrating substantial intra-district variation, this study supports the WHO recommendations calling for improved mapping and surveillance to optimize PC delivery, thereby reducing over-treatment in low-risk areas and under-treatment in transmission hotspots [28, 29]. Although fine-scale epidemiological assessments such as this are not routinely feasible in most programmatic contexts, the findings highlight the need for well-designed impact assessment surveys that can generate actionable data at appropriate geographic scales. Such data would enable national NTD programs to periodically reassess the spatial units used for PC implementation and to adopt more targeted treatment strategies in heterogeneous settings, consistent with national program priorities and the WHO 2030 goal of eliminating schistosomiasis as a public health problem [27].

There are several limitations to consider in this study. One is that the average number of children per household found in some villages was lower than the target value expected. This study took place during July and August, which are rainy periods and times of intense farming activity, leading to many absences from households. In addition, the survey was designed to optimize geostatistical models across the study area and was therefore not powered to provide precise empirical point prevalence estimates at each survey site. Due to the absence of available community sampling frames, simple random sampling within primary sampling units (PSUs) was not feasible. Instead, field teams used a modified compact segment sampling approach—a practical alternative that minimizes bias and maintains the representativeness of the survey area while being more operationally feasible. Urine filtration has low sensitivity, particularly for detecting low-intensity infections. This may have resulted in an underestimation of the prevalence of light infections in survey communities.

The results of this study will be used to guide current PC strategies in these districts but may also shed light on the heterogeneity of *S. haematobium* distribution that might be expected in settings with similar epidemiological profiles of schistosomiasis. To achieve Togo’s elimination goals, complementary strategies including awareness-raising for health behavior change, the provision of safe water and adequate sanitation facilities, and schistosomiasis control activities should be implemented simultaneously.

## Data Availability

Data supporting the main conclusions of this study are included in the manuscript.

## Abbreviations

PC: Preventive chemotherapy
SCH: Schistosomiasis
NTDs: Neglected tropical diseases
PHU: Peripheral Health Unit
STH: Soil-transmitted helminthiasis
MDA: Mass drug administration
PSU: Primary sampling unit
SAC: School-aged children
CHWs: Community health workers
SDK: Secure Data Kit platform
WASH: Water, Sanitation, and Hygiene
CBRS: Comité Bioéthique pour la Recherche en Santé
MSHP: Ministry of Health and Public Hygiene
